# Case report: Significance of the large rhomboid lip in microvascular decompression: insights from two clinical cases

**DOI:** 10.3389/fneur.2023.1336273

**Published:** 2024-01-16

**Authors:** Feiyu Ding, Pan Li, Xiaozhou Zuo, Wenxiong Song, Yong Xiao, Dong Wang, Liangyuan Geng, Xinhua Hu, Kun Yang, Yong Liu, Yuanjie Zou

**Affiliations:** ^1^Department of Neurosurgery, Affiliated Nanjing Brain Hospital, Nanjing Medical University, Nanjing, China; ^2^Department of Neurosurgery, Beijing Tiantan Hospital, Capital Medical University, Beijing, China; ^3^China National Clinical Research Center for Neurological Diseases, Beijing, China

**Keywords:** rhomboid lip, microvascular decompression, hemifacial spasm, glossopharyngeal neuralgia, flocculus

## Abstract

The rhomboid lip (RL) is a layer of neural tissue that extends outside the fourth ventricle and is connected to the lateral recess of the fourth ventricle. Although this anatomical structure has been rigorously studied, it is often overlooked in microvascular decompression (MVD) surgery. In this report, we present two cases, one of hemifacial spasm (HFS) and one of glossopharyngeal neuralgia (GPN), in which a large RL was observed during surgery. We found that a large RL is easily confused with arachnoid cysts, and accurate identification and dissection are important to protect the lower cranial nerves.

## Introduction

Microvascular decompression surgery is an effective treatment for hemifacial spasm (HFS) and glossopharyngeal neuralgia (GPN). Using the lateral suboccipital infrafloccular approach, the culprit vessels can be identified and isolated, which results in satisfactory outcomes. In this approach, the choroid plexus protruding from the foramen of Luschka serves as a significant anatomical landmark to locate the root exit zone (REZ) (1). The choroid plexus and the lower cranial nerves can be adequately visualized by gently retracting the flocculus posterosuperiorly. The rhomboid lip (RL) is sometimes encountered during dissection; however, it is sometimes discovered under normal circumstances (2–5). In this study, we report two microvascular decompression (MVD) cases with a large RL.

## Case presentation

### Case 1

**Figure 1:**
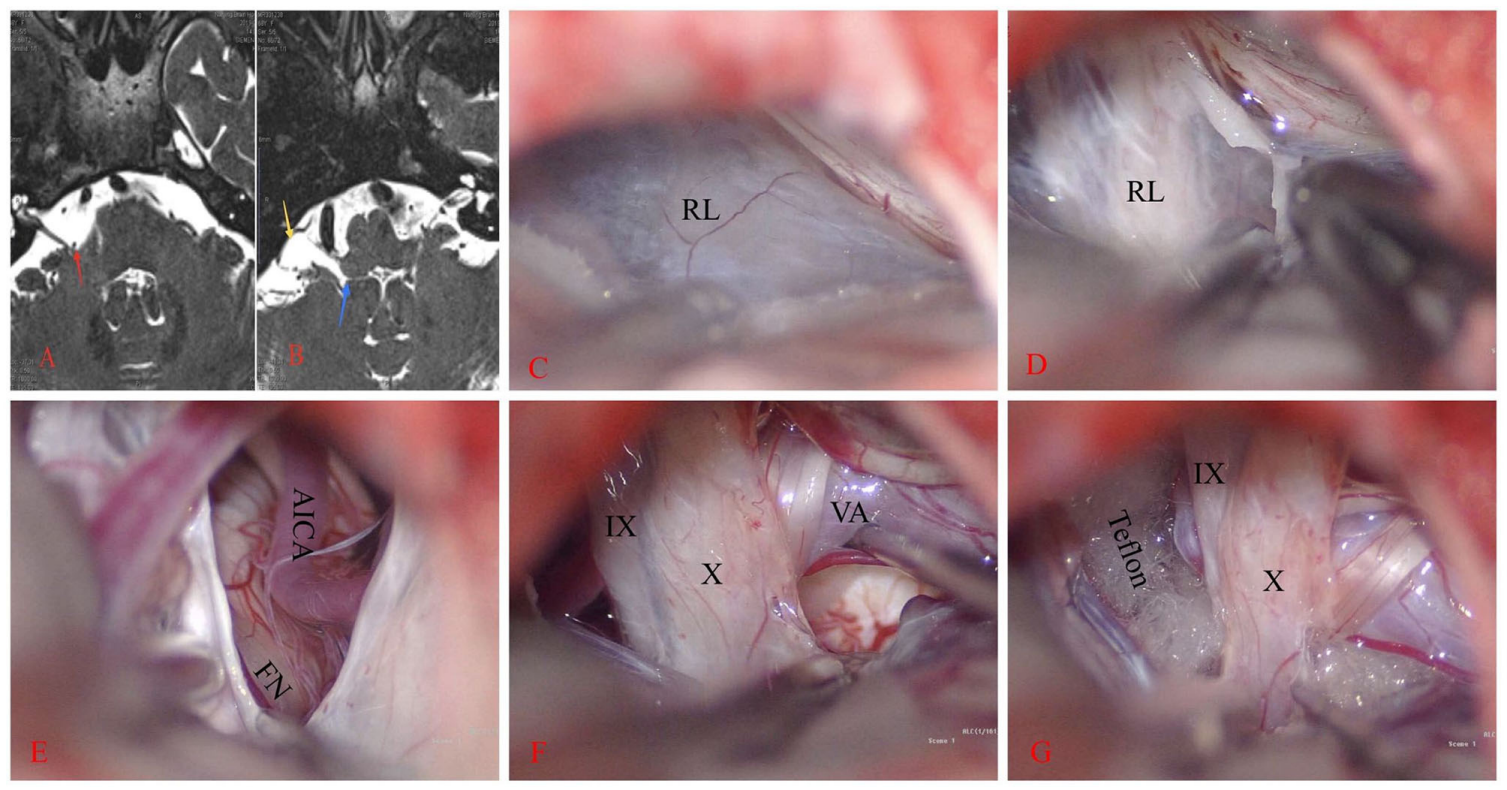
Right hemifacial spasm. **(A)** The anterior inferior cerebellar artery loop (AICA) compresses the facial nerve REZ (red arrow). **(B)** A sac-like structure on the ventral side of the flocculus (yellow arrow) is observed on T2WI. This structure communicates inwardly with the fourth ventricle through the foramen of Luschka (blue arrow). **(C, D)** A large rhomboid lip (RL) wraps around the jugular foramen and combines with the choroid to form a large lacuna. CN IX and X are located ventral to it and stick tightly. **(E–G)** After the RL is released, the vertebral artery (VA) and AICA are lifted away, revealing the REZ of the facial nerve (FN). Teflon is then placed between them.

A 68-year-old female was admitted to the hospital due to involuntary contraction at the corners of the right eye and mouth for over 3 years. Magnetic resonance imaging (MRI) revealed that the right anterior inferior cerebellar artery (AICA) formed a vascular loop and compressed the REZ of the facial nerve (Figure 1A). Additionally, a cystic structure was observed adjacent to the choroid plexus and connected medially to the foramen of Luschka (Figure 1B).

Once contraindications to surgery were excluded, the patient underwent MVD surgery under general anesthesia. Intraoperative electrophysiological monitoring was employed. The patient was positioned in the lateral park bench position. The dura was incised in a “C” shape and then suspended. Intraoperatively, a white translucent membranous cystic RL structure with tight adhesions to the lower cranial nerves was observed (Figures 1C, D). After excision and release of the RL, the lateral suboccipital infrafloccular approach was used to explore the REZ of the facial nerve. It was observed that the right AICA was ascending and compressing the REZ of CN VII (Figure 1E). Following the dissection of the arachnoid, a Teflon felt pledget was inserted (Figures 1F, G), resulting in the immediate disappearance of the LSR waveform during electrophysiological monitoring.

After the surgery, the patient's symptoms on the right side of the face disappeared. There were no observed cases of hearing impairment, facial paralysis, or recurrence during the 1-year follow-up period.

### Case 2

**Figure 2:**
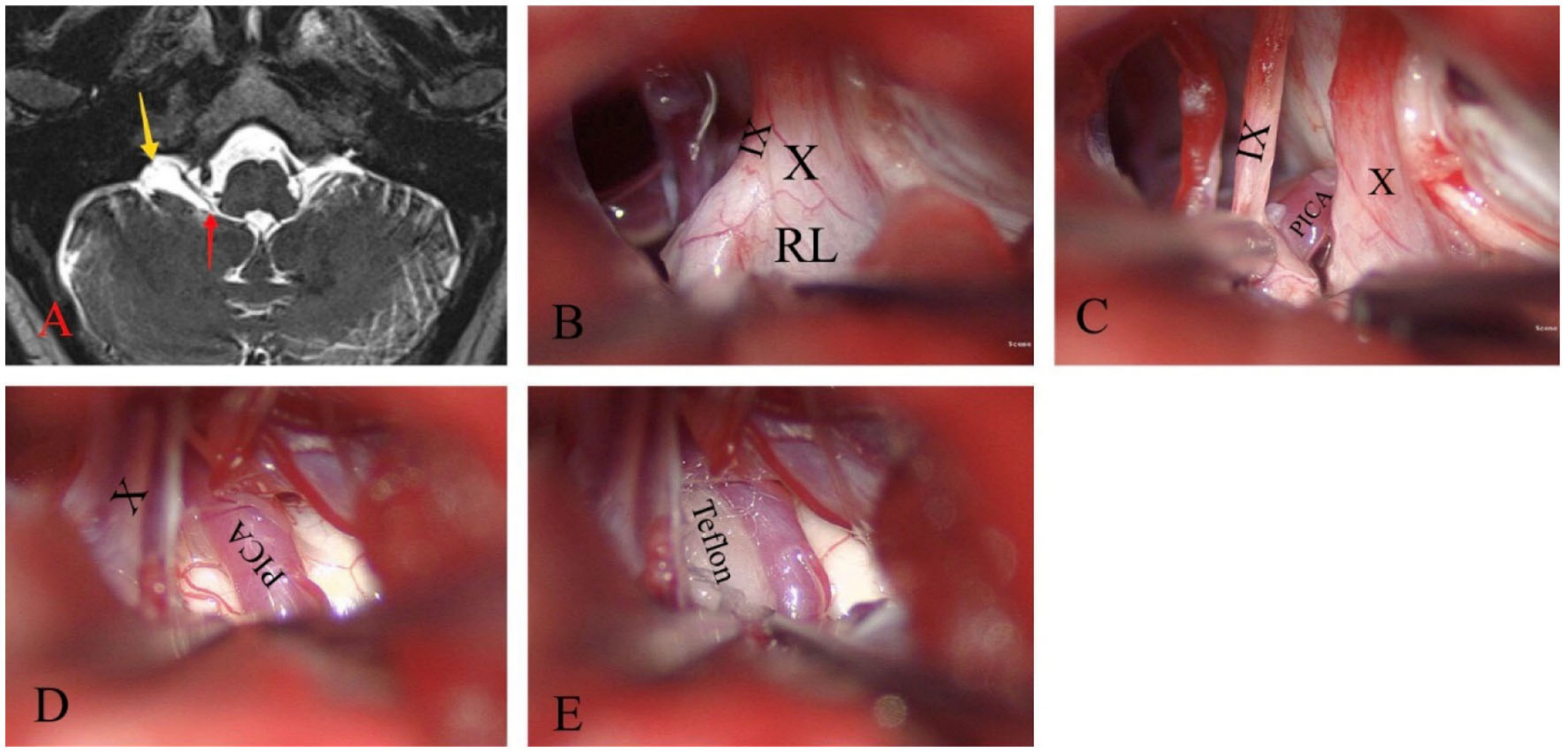
Glossopharyngeal neuralgia on the right side. **(A)** T2WI shows a sac-like structure (yellow arrow) located ventral to the flocculus that connects to the fourth ventricle through the foramen of Luschka (red arrow). **(B)** The large RL surrounds the jugular foramen and tightly adheres to the lower cranial nerves. **(C–E)** By releasing and resecting most of the RL, CN IX, CN X, and the posterior inferior cerebellar artery (PICA) were effectively exposed to compress the REZ. Teflon is then inserted between them.

A 66-year-old female was admitted to the hospital with a history of episodic right-sided pharyngeal pain lasting for more than 5 years. The findings from MRI T2WI and 3D-TOLF revealed a close relationship between the right posterior inferior cerebellar artery (PICA) and the glossopharyngeal nerve. The preoperative procaine test yielded positive results. Additionally, the MRI T2WI showed the presence of a sac-like structure positioned ventrally to the choroid and connected medially to the foramen of Luschka (Figure 2A).

A standard retrosigmoid suboccipital approach was performed using intraoperative neurophysiological monitoring. The infrafloccular approach was used to release the lower CNs. A milky white translucent membranous cystic RL was observed wrapping around the jugular foramen area and tightly adhering to the lower CNs (Figure 2B). After excision and dissection of the RL, the right PICA was found to be ascending and exerting pressure on the REZ of CN IX (Figures 2C, D). To alleviate this pressure, the PICA was liberated and a Teflon spacer was inserted (Figure 2E).

The patient experienced a resolution of symptoms, with no recurrence during a follow-up period of over 1 year.

## Discussion

Since the introduction of MVD by Jannetta for the treatment of cranial nerve disorders, it has become the mainstay of treatment for HFS and GPN (6–9). Although this technique is well-established, the literature reports an incidence of 0.5–3% for permanent cranial nerve dysfunction, such as facial palsy (10), hearing loss (11), and lower cranial nerve deficits (12, 13). These complications may be caused by intraoperative malpractice, as well as the complexity and variability of individual anatomy, which increases the difficulty of surgery. Furthermore, inadequate knowledge and mishandling of the RL can contribute to nerve injuries. The RL is occasionally mistaken for thickened arachnoid membranes, which increases the risk of cranial nerve injuries during separation. Therefore, it is vital to clearly understand the anatomical relationship between the arachnoid membrane, RL, and the lower CNs to ensure the safety of MVD.

**Figure 3:**
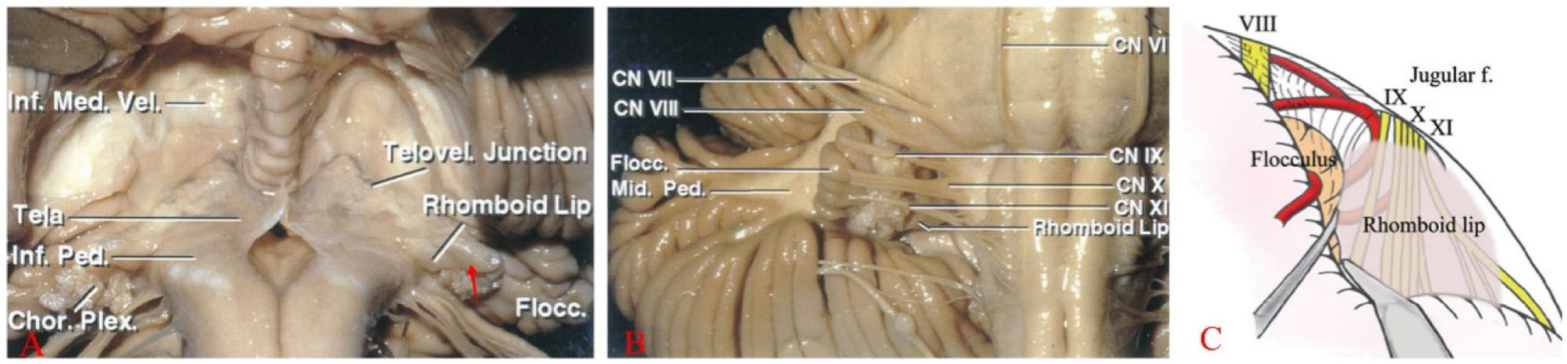
**(A)** The RL, a thin membranous neural structure, extends laterally from the floor of the fourth ventricle to join with the choroid plexus (Tela), forming a lacuna (red arrow) outside the lateral recess and the foramen of Luschka. **(B)** Located posterior to CNs IX, X, and XI, the RL is ventral to the recess and extends outward with the choroid plexus. **(C)** A large RL overlays the surface of the lower cranial nerves, obstructing the view of the REZ of CN VII and IX from the rear (Note: **A, B** are reproduced from *Cranial anatomy and surgical approaches* by Rhoton AL; Lippincott Williams & Wilkins, 2008. **C** is reproduced from Matsushima T. (ed): *Microsurgical Anatomy and Surgery of the Posterior Fossa*; Springer, 2015.).

As the fourth ventricle migrates into the lateral recess, the floor of the fourth ventricle and the RL form the ventral wall of the lateral recess (14). The RL continues to migrate ventrally. Along with the choroid plexus, the RL forms the most lateral end of the lateral recess, creating a trap-like structure (Figure 3A). At the lateral end, the RL forms the ventral margin of the foramen of Luschka, while the choroid and its accompanying choroid plexus form the dorsal margin. CN IX and CN X are located ventrally to the RL, with the CN VII and CN VIII and the choroid located anteriorly above it (Figures 3B, C). Akiyama et al. (15) classified RL into three types: non-extension type, lateral extension type, and jugular foramen type, based on the relationship of the RL to the choroid plexus. All types of RL surround CN IX and CN X and are in contact with or locally adherent to them. The jugular foramen type adheres extremely closely to CN IX.

Although researchers have provided detailed descriptions of the microanatomy of RL, the clinical significance of RL has not yet been recognized (16). This lack of recognition may be attributed to the inadequate understanding of RL anatomy and structure. In a study conducted by Nakahara et al. (4), only 9 out of 34 patients with HFS had visualized RL during surgery (26.5%), and only 3 cases (8.8%) could be demonstrated on preoperative MRI. On T2WI MRI, a larger RL exhibited high signal cystic manifestations ventral to the flocculus, which connected medially with the fourth ventricle through the foramen of Luschka. However, visualizing smaller RLs was challenging. A case study by Cho et al. (3) reported the misdiagnosis of a large RL as an arachnoid cyst, which highlights the importance of understanding RL anatomy and features to avoid such misdiagnoses. RL and arachnoid cysts are distinct tissue structures. The RL is visually thicker, translucent, and milky white compared to the arachnoid. Histologically, the RL has a two-layered structure with ciliated cells in the inner layer, similar to the ventricular cells at the base of the fourth ventricle, and an outer layer that consists of glial cells and neurons. In contrast, the arachnoid membrane lacks nerve cells. Anatomically, the outer arachnoid membrane accompanies the lower CNs into the jugular foramen, whereas the RL does not enter the jugular foramen.

In MVD surgery, it is important to release and separate the RL in a precise manner. Precision separation allows the surgeon to clearly view the REZ of CN VII, IX, and X while avoiding any unnecessary retraction of the lower CNs. By gently retracting the flocculus posterosuperiorly using the infrafloccular approach, the RL and the REZ of CN VII can be sufficiently visualized. However, if the RL is large or tightly adherent to CN IX and X, it becomes difficult to visualize the REZ of CN VIII, IX, and X. Improper retraction or failure to correctly identify the relationship between the RL and the lower nerves can also lead to postoperative cranial nerve injury. In patients with a large RL, considering its anatomical relationship with CN VII and IX is important. To avoid injury to the lower CNs, the RL should be patiently and carefully dissected from these cranial nerves or separated in the direction of the nerve alignment before retracting the flocculus. This allows for improved visualization of the periphery of the REZ of CN VII, IX, and X and the brainstem.

## Conclusions

A thorough anatomical knowledge and meticulous dissection can help surgeons observe the structures of the foramen of Luschka region more clearly. These practices can ensure the protection of cranial nerves, thereby reducing the risk of cranial nerve injuries.

## Data availability statement

The original contributions presented in the study are included in the article/supplementary material, further inquiries can be directed to the corresponding author/s.

## Ethics statement

The studies involving humans were approved by Medical Ethics Committee of Nanjing Brain Hospital. The studies were conducted in accordance with the local legislation and institutional requirements. Written informed consent for participation was not required from the participants or the participants' legal guardians/next of kin in accordance with the national legislation and institutional requirements. Written informed consent was obtained from the individual(s) for the publication of any potentially identifiable images or data included in this article.

## Author contributions

FD: Writing – original draft, Writing – review & editing. PL: Data curation, Writing – review & editing. XZ: Data curation, Writing – review & editing. WS: Data curation, Investigation, Writing – review & editing. YX: Investigation, Supervision, Writing – original draft. DW: Investigation, Supervision, Validation, Writing – review & editing. LG: Supervision, Validation, Writing – review & editing. XH: Supervision, Validation, Writing – review & editing. KY: Supervision, Validation, Writing – review & editing. YL: Supervision, Validation, Writing – review & editing. YZ: Funding acquisition, Supervision, Validation, Visualization, Writing – review & editing.
